# Evaluation of a Prototype for Electrochemical pH-Shift Crystallization of Succinic Acid

**DOI:** 10.3390/ma15238412

**Published:** 2022-11-25

**Authors:** Christian Kocks, Diana Wall, Andreas Jupke

**Affiliations:** AVT—Fluid Process Engineering, RWTH Aachen University, Forckenbeckstraße 51, 52074 Aachen, Germany

**Keywords:** electrochemical crystallization, succinic acid, electrified downstream processing, pH-shift crystallization

## Abstract

Downstream processing of biotechnologically produced carboxylic acids, such as succinic acid, poses environmental and economic challenges. Conventional downstream processes cause large amounts of waste salts, which have to be purified or disposed of. Therefore, lean and waste-free downstream processes are necessary for the biotechnological production of succinic acid. Electrochemical downstream processes gain especially significant attention due to low chemical consumption and waste reduction. This work presents the pH-dependent solid-liquid equilibrium of succinic acid, a prototype for electrochemical pH-shift crystallization processes, and its characterization. Based on the supersaturation, energy consumption, and electrochemical protonation efficiency the proposed electrochemical pH-shift crystallization is evaluated. This evaluation highlights the potential of the proposed electrochemical crystallization processes as waste-free and economically attractive processes for bio-based succinic acid production.

## 1. Introduction

Bio-based carboxylic acids, like succinic acid (SA), offer strong potential as future platform chemicals [1]. The development of highly productive microorganisms enabled large-scale commercial succinic acid production, competing with conventional petrochemical production [2,3,4]. Nevertheless, bio-based succinic acid is rarely used as an intermediate in the chemical industry due to its high production costs caused by high raw material costs for biotechnological production, and expensive and challenging downstream processing [4,5,6]. Reported downstream processes for succinic acid consist of a combination of extraction, adsorption, electrodialysis, precipitation and crystallization steps [3,5,6,7]. Most of these processes rely on the costly addition of strong acids and bases for the pH-management during recovery and purification of succinic acid [5,7]. Additionally, the use of pH-adjusting agents leads to the co-production of salts, such as gypsum (CaSO_4_), which results in further costs for disposal or treatment. Therefore, downstream processing of succinic acid has to become more economical and ecological by avoiding pH-adjusting agents.

A promising concept to avoid waste salt production is that of electrified downstream processes, which use water-splitting electrolysis in order to control and adjust the pH value [8,9,10,11,12]. The electrification of downstream processes has gained significant interest and has shown promising results for the purification or in situ separation of carboxylic acids [10,11,12,13,14,15,16,17]. Urbanus et al. [8] demonstrated the feasibility of electrochemically induced crystallization for in situ product removal of carboxylic acids. Furthermore, several works illustrate the synergies between electrified downstream processes and pH-control of fermentation processes [8,9,10,11]. However, these processes usually can not compete with conventional processes due to high operation costs or low stability of the membranes due to scaling or fouling [5,18]. In previous work, we showed that crystallization on the membrane can be prevented and pure succinic acid can be produced via electrochemical pH-shift crystallization [19,20,21]. However, we could not reach high current densities due to high electrical resistances within the reactors used for the feasibility studies, which led to slow crystallization [19,20,21].

To overcome these limitations, a prototype of an anode chamber was designed, which allows stable operation of the electrochemical pH-shift crystallization. This work aims to present and characterize the prototype’s performance for the electrochemical pH-shift crystallization process presented in previous works [19,20,21]. Within these works, the feasibility of the electrochemical pH-shift crystallization was shown and the nucleation mechanism close to the anode was investigated. However, the electrochemical pH-shift crystallization was not operated at industry-relevant operation conditions. The designed prototype enables these operation conditions, by reducing ohmic resistance, and allows an assessment of the potential of the electrochemical pH-shift crystallization as a downstream process for bio-based succinic acid. Therefore, the behavior of the prototype is evaluated with respect to selected key operating conditions such as temperature and current density. Finally, this work allows an estimation of the operating costs for pH-management of the envisaged electrochemical pH-shift crystallization of bio-based succinic acid.

### 1.1. Electrochemical pH-Shift Crystallization

The driving force for the electrochemical pH-shift crystallization is generated via water-splitting electrolysis. At the anode, water is split into oxygen and protons (Equation (1)), while the cathode reaction produces hydrogen and hydroxide ions (Equation (2)).

(1) $$2H_{2}O\to O_{2}+4H^{+}+4e^{-}$$

(2) $$4H_{2}O+4e\to 2H_{2}+4OH^{-}$$

The protons remain in the vicinity of the anode, if additional positively charged ions, such as Na^+^, can migrate through the cation exchange membrane between the anode and cathode. Hence, the concentration of protons increases at the anode, which leads to a decrease of the pH value (Equation (3)):

(3) $$\mathrm{pH}=-\log_{10}\left(c_{\left(H^{+}\right)}\right).$$

The shift of the pH value is induced by the amount of protons generated by water-splitting electrolysis $n_{H^{+},\mathrm{el}}$, which depends on the electric current I, the time t, z the number of electrons transferred in the electrochemical reaction and the Faraday constant F (Equation (4)).

(4) $$n_{H^{+}\mathrm{el}}=\frac{I\;t}{z\;F}=\frac{Q}{z\;F}$$

Thus, the pH value can be controlled by the electric charge Q introduced into the aqueous system. Depending on the pH value, succinic acid is protonated (H_2_SA) or dissociated (HSA^−^ or ${\mathrm{SA}}^{2-}$) in the aqueous solution. The dissociation equilibrium of succinic acid is defined by the dissociation constants ${\mathrm{pK}}_{a1}=3.96$ and ${\mathrm{pK}}_{a2}=5.28$ [22].

(5) $$H_{2}SA⇌HSA^{-}+H^{+}\;K_{a1}=\frac{c_{\left(H^{+}\right)}\cdot c_{\left({\mathrm{HSA}}^{-}\right)}}{c_{\left(H_{2}SA\right)}}$$

(6) $${\mathrm{HSA}}^{-}⇌{\mathrm{SA}}^{2-}+H^{+}\;K_{a2}=\frac{c_{\left(H^{+}\right)}\cdot c_{\left({\mathrm{SA}}^{2-}\right)}}{c_{\left({\mathrm{HSA}}^{-}\right)}}$$

The knowledge of the dissociation equilibrium and the pH value allows us to calculate the relative amount of the different succinic acid species (α):

(7) $$\alpha_{0}=\frac{c_{\left(H^{+}\right)}^{2}}{c_{\left(H^{+}\right)}^{2}+K_{a1}\;\cdot \;c_{\left(H^{+}\right)}+K_{a1}\;\cdot \;K_{a2}}=\frac{c_{\left(H_{2}\mathrm{SA}\right)}}{c}$$

(8) $$\alpha_{1}=\frac{c_{\left(H^{+}\right)}\;\cdot \;K_{a1}}{c_{\left(H^{+}\right)}^{2}+K_{a1}\;\cdot \;c_{\left(H^{+}\right)}+K_{a1}\;\cdot \;K_{a2}}=\frac{c_{\left({\mathrm{HSA}}^{-}\right)}}{c}$$

(9) $$\alpha_{2}=\frac{K_{a1}\;\cdot \;K_{a2}}{c_{\left(H^{+}\right)}^{2}+K_{a1}\;\cdot \;c_{\left(H^{+}\right)}+K_{a1}\;\cdot \;K_{a2}}=\frac{c_{\left({\mathrm{SA}}^{2-}\right)}}{c}.$$

**Figure 1:**
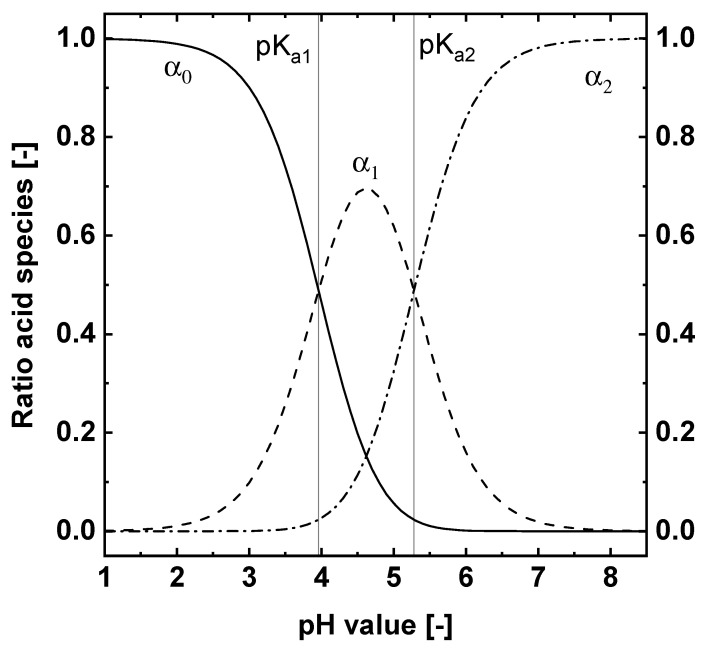
Dissociaction of succinic acid over the pH value calculated with the ${\mathrm{pK}}_{a1}=3.96$ and ${\mathrm{pK}}_{a2}=5.28$ reported by Arendt [22].

$\alpha_{0}$ is the ratio of the protonated species to the summation of all three species c, $\alpha_{1}$ and $\alpha_{2}$ the ratio of the dissociated species (Figure 1).

Hence, the amount $n_{H^{+},\mathrm{Diss}}$ of H^+^ ions necessary to reach the dissociation equilibrium at a certain pH can be calculated by Equations (3), (5)–(9). This amount can be used for an estimation of the protonation efficiency η:

(10) $$\eta =\frac{n_{H^{+},\mathrm{Diss}}}{n_{H^{+},\mathrm{El}}}.$$

The molar amount of H^+^ ions produced by water splitting calculated by Faraday’s law (Equation (4)) is represented as $n_{H^{+},\mathrm{El}}$. η shows how much of the electrochemically produced protons are used for the protonation of succinic acid.

The dissociation equilibrium shifts towards the protonated species of succinic acid by decreasing the pH value (Figure 1). Below a pH value of approx. 4.4, the protonated succinic acid (H_2_SA) can crystallize if the concentration is high enough and the system is supersaturated (S > 1 Equation (11)) [23]. The supersaturation ratio S for the summation of the succinic acid species at a given pH value is defined by:

(11) $$\sigma =\frac{\sum_{i=0}^{3}c_{i}-\sum_{i=0}^{3}c_{i}^{*}}{\sum_{i=0}^{3}c_{i}^{*}}=\frac{\left(c-c^{*}\right)}{c^{*}}=S-1.$$

c is the measured molar concentration of all three succinic acid species combined at a given pH, σ is the relative supersaturation and $c^{*}$ is the solubility concentrations of the three species.

### 1.2. Electrified pH-Shift Downstream Process for Bio-Based Succinic Acid

**Figure 2:**
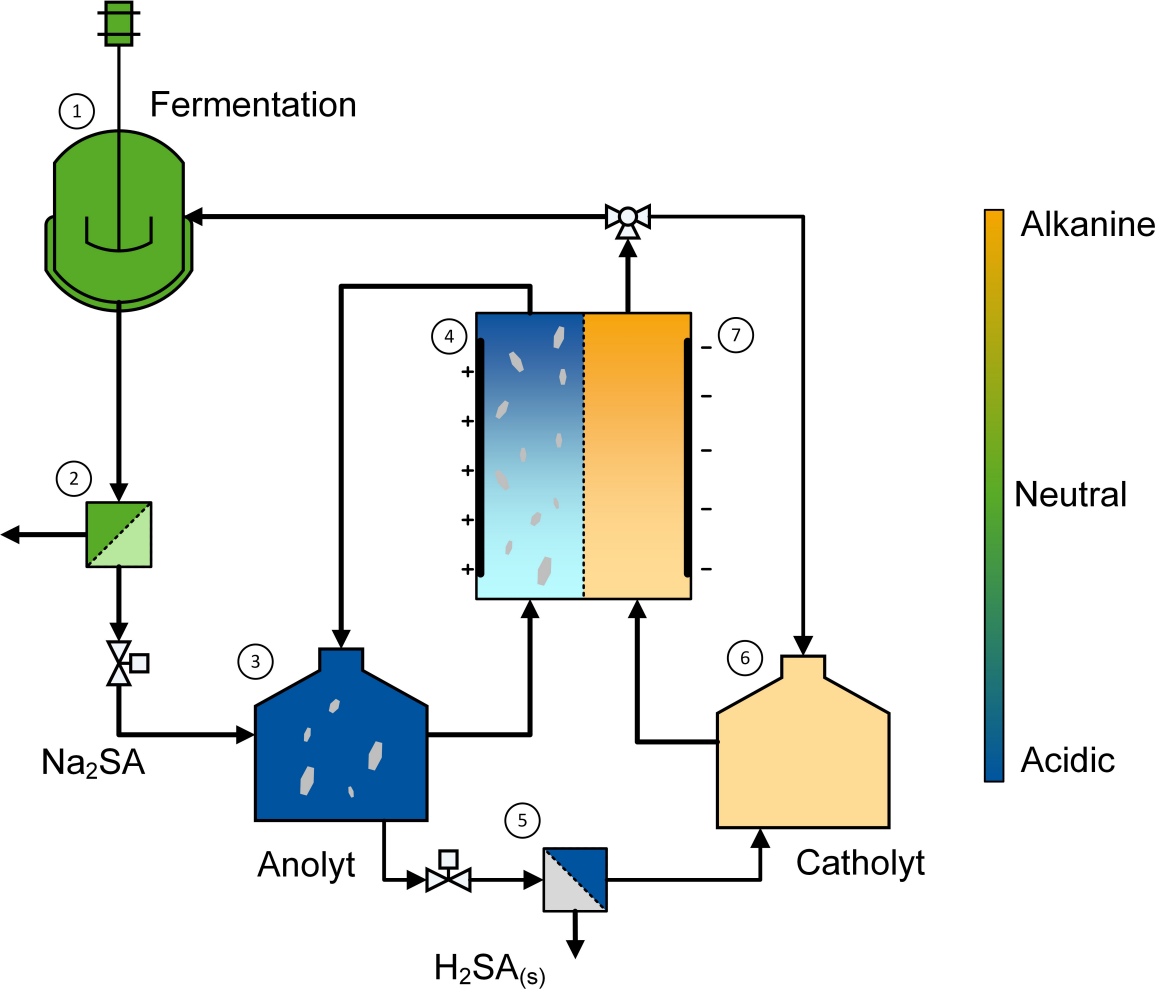
Electrified pH-shift downstream process for bio-based succinic acid adapted from Kocks et al. [19]: (1) fermentation at neutral pH value, (2) cell removal, (3) anolyt reservoir, (4) electrochemical acidification and pH-shift crystallization, (5) succinic acid crystal recovery, (6) catholyte reservoir and (7) electrochemical basification for pH-management in the fermentation.

Figure 2 shows the electrochemical pH-shift crystallization embedded into the entire process concept [19]. After filtration (2), the cell-free fermentation broth (1) is fed into the anolyte tank. In the anolyte tank (3), the pH value is kept within the pH-region for the pH-shift crystallization by the water-splitting reaction (Equation (1)) taking place in the anode chamber of the electrolysis cell (4). In the anode chamber (4), high volume flows are necessary in order to avoid sedimentation of the crystals. These volume flows do not allow a full protonation of high succinic acid concentration in one passage of the anode chamber. That is why a loop between the anolyte tank (3) and the anode chamber (4) is implemented. The produced crystals can be recovered by solid–liquid separation (5) and the mother liquid is recycled into the catholyte tank (6). Simultaneously, the pH value is increased in the catholyte tank due to the cathode reaction (7) (Equation (2)), which produces an alkaline solution, which can be recycled to the fermenter (1) and substitute base consumption during microbial succinic acid production.

## 2. Materials and Methods

### 2.1. Solubility-Measurements

In order to evaluate the crystallization process, especially the supersaturation and the yield of the crystallization experiments, the knowledge of the solubility curve for succinic acid is essential [24,25]. It defines the amount of dissolved succinic acid at a given pH value, temperature, and composition of the solution. Therefore, the solubility of succinic acid was measured for relevant temperatures and pH values in aqueous solutions. 0.5 mol L^−1^ disodium sulfate (Na_2_SO_4_) was added to the aqueous phase since it is used as background electrolyte in the crystallization process for the water-splitting electrolysis. The solubility concentrations were determined at 10 °C and 30 °C in aqueous Na_2_SO_4_ solutions. The excess method was chosen for the determination of the solubility, where a solid phase always remains within the samples in order to ensure a saturated aqueous solution [20]. The pH value was adjusted by succinic acid to disodium succinate ratio. This ratio was adapted in order to keep the pH value lower than 4.4, making only the crystallization of succinic acid possible [23] due to the high solubility of the sodium salts of succinic acid. The samples were tempered and mixed for at least 24 h. Afterwards, the pH value of the solution and the succinic acid concentration were measured.

In order to determine the concentration of succinic acid, samples were drawn with a syringe filter Chromafil Xtra H-PTFE-20/13 by Macherey-Nagel (Düren, Germany). The samples were diluted with distilled water in a ratio of 1:4. The concentration of succinic acid in each sample was determined by an Agilent (Ratingen, Germany) 1100 series HPLC, equipped with an organic acid resin column (250 × 8 mm, LC-OrganicAcid-CS-S, CS Chromatography, Langerwehe, Germany) operated at 40 °C. A refractive index detector (RID) G1362A was used and the elution was carried out with a 10 mM aqueous sulfuric acid solution with a flowrate of 0.7 mL min^−1^. Sample injection volume was 5 μL. The pH value was measured with the FiveEasy pH meter by Mettler Toledo (Columbus, OH, USA).

### 2.2. Prototype

**Figure 3:**
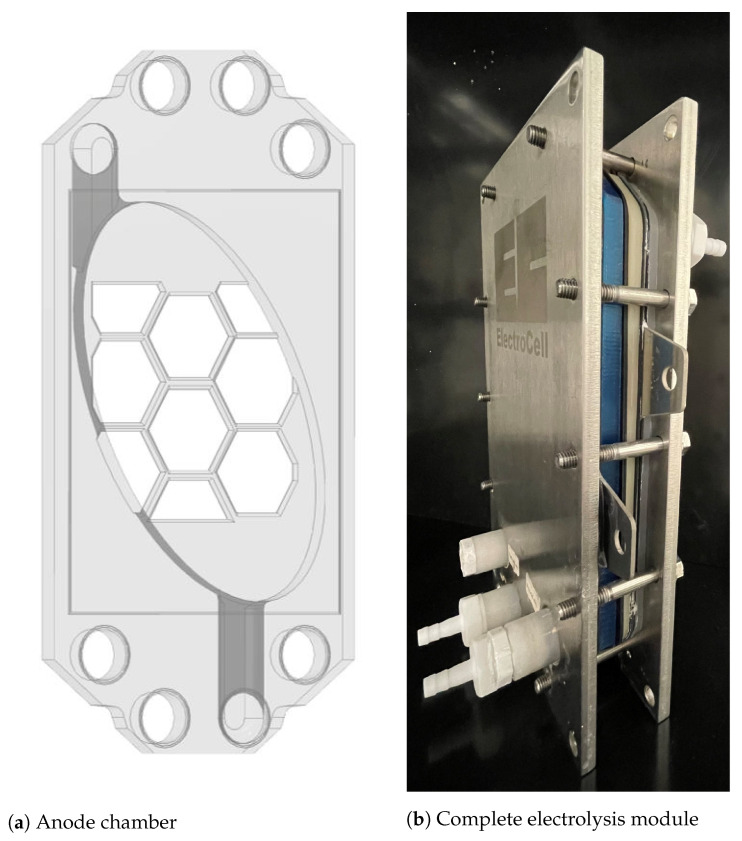
Drawing of the prototype for the electrochemical pH-shift crystallization on the left and on the right the prototype of the anode chamber (blue) integrated in the frameworks of the Electro MP Cell^®^.

The prototype of the anode chamber shown in Figure 3a for the electrochemical pH-shift crystallization was manufactured by Fused Deposition Modeling 3D-Printing. The prototype is made of PETG, which is a glycol-modified PET (polyethylene terephthalate) polymer. It was designed in such a way that it works within the frameworks of the Electro MP Cell^®^ (ElectroCell A/S, Tarm, Denmark), which is an electrolysis module with an electrode surface of 100 cm${}^{2}$ (Figure 3b). This allows fast adaptations of the prototype as well as ensures a standardized electrolysis setup. In order to minimize stagnation zones and simultaneously maximize the effective electrode area, an oval form was chosen. The prototype has a depth of 8 mm and a total volume of approx. 0.12 L for the suspension flow. Within these 8 mm, a honeycomb structure of 2 mm was designed in order to stabilize the membrane (Figure 3a). Together with the 8 mm cathode chamber and the sealing, this leads to an electrode gap of 20 mm, excluding the membrane thickness. A Nafion 117 cation exchange membrane separates both chambers. The electrolysis cell was equipped with a ruthenium mixed metal-coated titanium anode and a nickel cathode.

### 2.3. Crystallization Experiments

Steps 3–7 of the envisaged electrified downstream process presented in Figure 2 were investigated in this study. The dosage of fresh fermentation broth (steps two to three) was simulated by using a pH-neutral disodium succinate solution and the acid recycled from steps 5 to 6 was realized by succinic acid addition in the catholyte tank. All experiments shown in this study were repeated at least twice. However, for clarity reasons, the results of one experiment are shown per figure.

The anolyte contained 0.78 mol L^−1^ disodium succinate, which is within the range of typical fermentation processes (between 0.4 mol L^−1^ and 1.2 mol L^−1^) [26], and 0.5 mol L^−1^ Na_2_SO_4_ as a background electrolyte. For the catholyte solution 0.42 mol L^−1^ succinic acid, which is the solubility of succinic acid at low pH at 20 °C and 0.5 mol L^−1^ (Table 1), Na_2_SO_4_ was used [20]. An initial volume of 0.5 L of the anolyte and 0.8 L of the catholyte were added to their respective tanks (Table 2). The used tanks were 1 L double-walled reaction vessels for temperature control, which were equipped with pH and temperature sensors.

Once the solutions reached the desired temperature, the experiment was started by switching on the power source (EA-PS9040-40T-640, Viersen, Germany) and the pumps (Ismatec MCP standard, Wertheim-Mondfeld, Germany). By turning the electricity on, the pH value decreased in the anolyte chamber and increased in the catholyte chamber during the entire experiment. The volume flow for both chambers was set to 0.52 L min^−1^, which corresponds to a space-time of $\tau =\frac{V}{\dot{V}}=5\;s$ in the anode chamber.

The pH-buffering ability of succinic acid ends at pH values above six. Exceeding this pH value, the catholyte quickly reaches the strongly alkaline pH region. In order to avoid an excessive difference in pH values between the two chambers and to account for the recycling of acidic solution within the process concept (steps five to six in Figure 2), the pH value of the catholyte was kept in the buffer range of the succinic acid throughout the whole experiment by adding 40 g of succinic acid as soon as the pH value of the catholyte chamber reached six. In contrast to that, the pH of the anolyte was controlled by adding 0.125 L of the initial anolyte solution to the anolyte chamber when a pH value of three was reached. This procedure was repeated three more times so that finally 1 L of the anolyte solution was processed during one experiment simulating the addition of fresh fermentation solution (Figure 2). The experiment ended as soon as a pH value of 2 was reached after the last dosage, since at this point all the succinic acid is protonated (Equations (5) and (6)). Once the experiment was completed, the anolyte was filtered using a vacuum pump to collect the generated crystals. Additionally, the electrolysis cell was opened in order to observe scaling or blocking of the prototype and scrape out the remaining crystals. All crystals were dried in a vacuum oven at 100 mbar and 50 °C for at least 12 hours. The dried crystals were analyzed with a Camsizer X2 by Retsch technology to determine the particle size distribution (compare Supplementary Materials). Furthermore, the crystals were analyzed by Raman spectroscopy (Rxn2—Endress Hauser) in order to detect possible impurities (compare Supplementary Materials). The concentration of succinic acid in the liquid phase of the anolyte and the catholyte was determined over the entire duration of the experiment. For this purpose, samples were taken and measured using the same procedure as mentioned above for the solubility measurements.

The electrochemical pH-shift crystallization was performed to investigate the impact of temperature and applied current density on the performance of the pH-shift crystallization (Table 3). Furthermore, Exp4 was performed in order to validate the findings of Exp1–Exp3. For the evaluation, the protonation efficiency of the succinic acid (Equation 10), the produced amount of succinic acid crystals, and the energy consumption during the crystallization were considered.

## 3. Results and Discussion

### 3.1. Solubility of Succinic Acid

**Figure 4:**
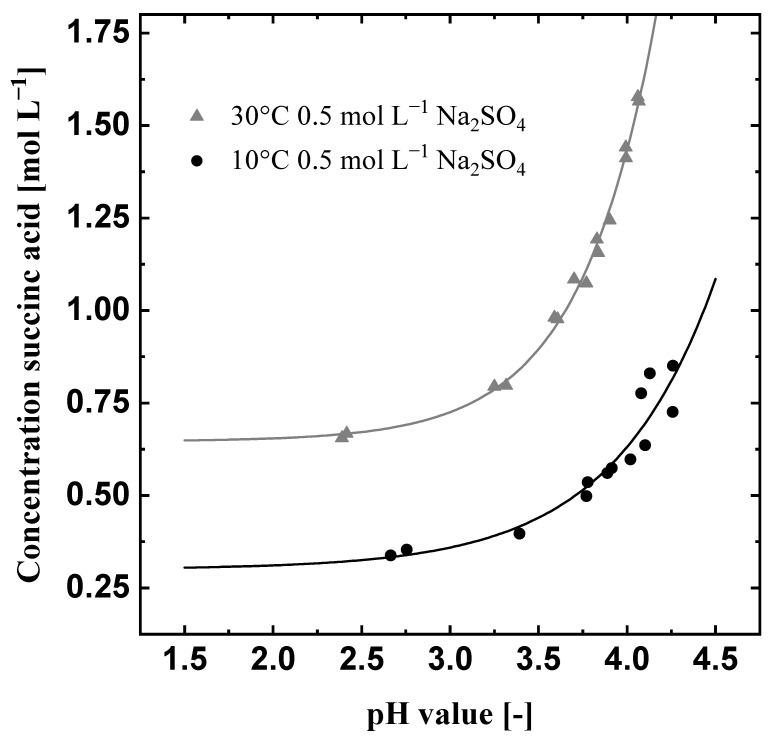
Influence of the pH value on the solubility concentration of succinic acid in aqueous Na_2_SO_4_ solutions at 10°C and 30°C.

Figure 4 displays the solubility curve of succinic acid at 10 °C and 30 °C in an aqueous Na_2_SO_4_ solution depending on the pH value. The solubility curve of succinic acid considering all three succinic acid species is shown in Figure 4 (Equation (11)).

The solubility concentration increases with the pH value. The rising solubility concentration is explained by the dissociation of succinic acid. At higher pH values a larger amount of dissociated succinic acid salts can be dissolved. In this case, the sodium salts of succinic acid are more soluble than the protonated succinic acid, which leads to overall higher solubility concentrations [20,23,27]. Furthermore, the solubility increases with rising temperatures, the difference in the solubility concentration between 10 and 30 °C at low pH values is more than 0.3 mol L^−1^. The measured solubility concentrations were fitted by the MATLAB Curve Fitting toolbox to the following equation:

(12) $$c^{*}=A_{\mathrm{SOL}}\;\cdot \;\exp^{\left(\mathrm{pH}\;\cdot \;B_{\mathrm{SOL}}\right)}+C_{\mathrm{SOL}}.$$

$A_{\mathrm{SOL}}$, $B_{\mathrm{SOL}}$ are fitting parameters, while $C_{\mathrm{SOL}}$ is the solubility concentration of the protonated acid [20,27] (Table 4).

### 3.2. Evaluation of Electrochemical pH-Shift Crystallization Experiments

**Figure 5:**
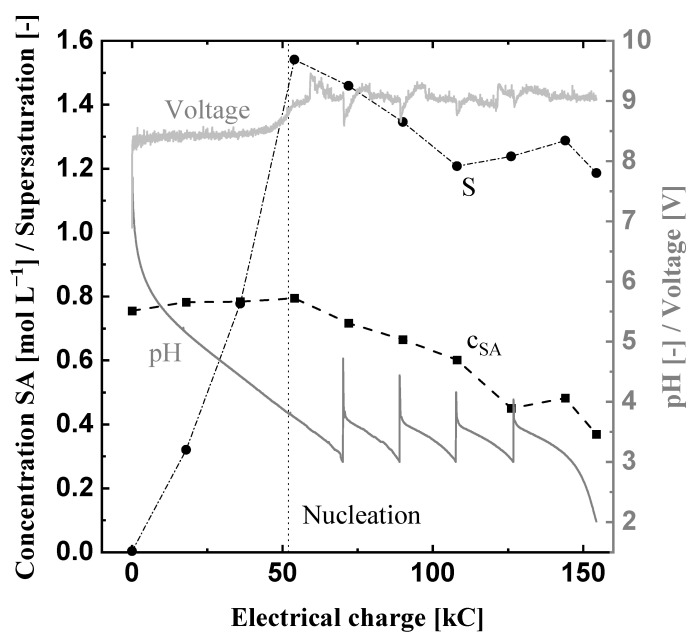
Electrochemical pH-shift crystallization in the prototype at 10 °C, 0.1 A cm^−2^ and a volume flow rate $\dot{V}$ of 0.52 L min^−1^ (Exp1). The black squares are the measured succinic acid concentration, the black cycles show the calculated supersaturation ratio S, and the two gray lines display the course of the pH value and voltage of Exp1.

Figure 5 shows the course of the combined succinic acid concentration of all three species in the prototype anode chamber, the calculated supersaturation ratio, the pH value, and the voltage of the electrolysis module over the introduced electric charge of Exp1.

The applied current density of 0.1 A c$m^{-}$${}^{2}$ leads to a duration of about 4.3 h at a temperature of 10 °C. With the increasing electrical charge, the pH value decreases from 7.8 to 3. At this point, the new pH-neutral succinic acid solution is fed into the anolyte tank, in order to simulate the feed of fresh fermentation broth (Figure 2). Hence, the pH value of the anolyte increases. These dosages are repeated four times until 1 L of the succinic acid solution is acidified to a pH value of two. Since the further reduction of the pH value will not reduce the solubility of succinic acid anymore (Figure 4), the experiment is stopped there. The initial succinic acid concentration stays constant for a third of the experiment’s duration and, after the nucleation, it decreases from about 0.8 mol $L^{-1}$ to 0.4 mol $L^{-1}$. Initially, the supersaturation ratio, calculated by Equation (11), rises until nucleation takes place and stays between 1.6 and 1.2 afterwards.

The current density is kept constant, hence the voltage changes due to variations in the conductivity of the solution or other additional electric resistances. In the beginning, the voltage amounts to roughly 8.5 V. When nucleation takes place, an increase in the cell voltage is observed. Most likely, this is caused by the formation of solid particles within the anode chamber generating an additional electrical resistance. Similarly, the two dosages of solid succinic acid in the catholyte, at about 60 kC and 120 kC, also result in two steep increases of the voltage. In contrast to that, the four dosages of pH-neutral disodium succinate solution result in a decrease of the voltage, due to the partial dissolution of the solid succinic acid crystals and the additional Na^+^ ions.

The final supersaturation ratio of about 1.2 indicates that the equilibrium is not reached at the end of Exp1. This leads to the assumption that the crystallization kinetics, which reduce the supersaturation (nucleation and growth), are slower than the supersaturation generation at the current density of 0.1 A cm${}^{-2}$. Nevertheless, 0.55 mol solid succinic acid crystals with a mean size $X_{50}$ of 478 μm (crystal size distribution in the Supplementary Materials) were recovered, which results in a specific energy demand of 0.69 kW h mol^−1^ produced succinic acid. The generated succinic acid crystals lead to a productivity for Exp1 of 0.13 mol L^−1^ h^−1^. Furthermore, the protonation efficiency (calculated with Equation (10)) amounts to roughly 98%, which shows that most of the electrochemically produced H^+^ ions are used as intended for the protonation of the succinic acid (Table 5).

**Figure 6:**
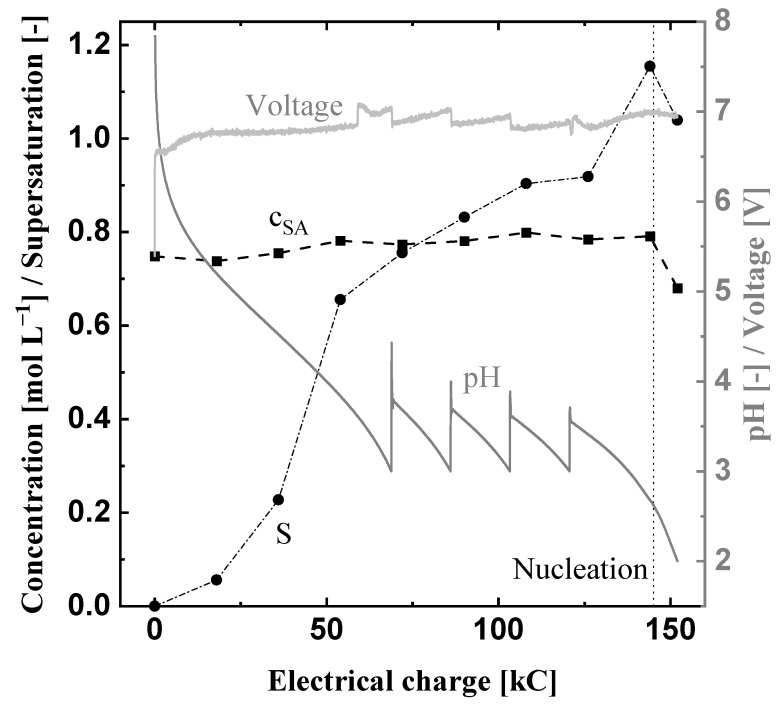
Electrochemical pH-shift crystallization in the prototype at 30 °C, 0.1 A cm^−2^ and a volume flow rate $\dot{V}$ of 0.52 L min^−1^ (Exp2). The black squares are the measured succinic acid concentration, the black cycles show the calculated supersaturation ratio S, and the two gray lines display the course of the pH value and voltage of Exp2.

In order to reduce the required energy, Exp2, shown in Figure 6, is conducted at 30 °C, since the conductivity increases with temperature. Except for the temperature, all operation parameters were the same as in Exp1 (Table 3). Figure 6 displays a reduction of the initial voltage by about two volts compared to Exp1 (Figure 6). The increases and decreases of the voltage due to the dosages in the anolyte and catholyte are comparable to Exp1. However, the nucleation takes place much later at the end of the experiment at 140 kC and does not result in a significant voltage increase. The pH value within the prototype shows a similar behavior at 30 °C as it did at 10 °C (Exp1). The initial succinic acid concentration of 0.78 mol L^−1^ stays constant and the supersaturation ratio stays below 1 until nucleation occurs. Afterwards, the concentration is reduced to about 0.7 mol L^−1^ while the supersaturation ratio lies between values of 1.2 and 1.04. The higher solubility concentration at 30 °C leads to smaller supersaturation ratios since the initial concentration was kept constant for all experiments. Nevertheless, the system does not reach equilibrium at the end of the crystallization experiment according to the solubility concentration measured in this work (Figure 4). Similar to Exp1, this leads to the assumption that the supersaturation at 0.1 A cm^−2^ is generated faster than it can be reduced due to the growth and nucleation of succinic acid crystals.

Due to the higher temperature, the voltage and the resulting energy demand were reduced from 0.38 kWh in Exp1 to 0.29 kWh in Exp2. The protonation efficiency is comparable to Exp1 (Table 5), which indicates that, for the full protonation of succinic acid (pH = 2), effects of the temperature on the protonation efficiency are negligible. However, less succinic acid was produced since the solubility is significantly higher at 30 °C (Figure 4). The product loss increased the specific energy demand to 1.89 kW h mol^−1^ and also reduced the productivity to 0.04 mol L^−1^ h^−1^. Thus, higher temperatures allow the reduction of the required energy for the pH-shift but the final temperature for the electrochemical pH-shift crystallization should be as low as possible for high succinic acid recovery.

**Figure 7:**
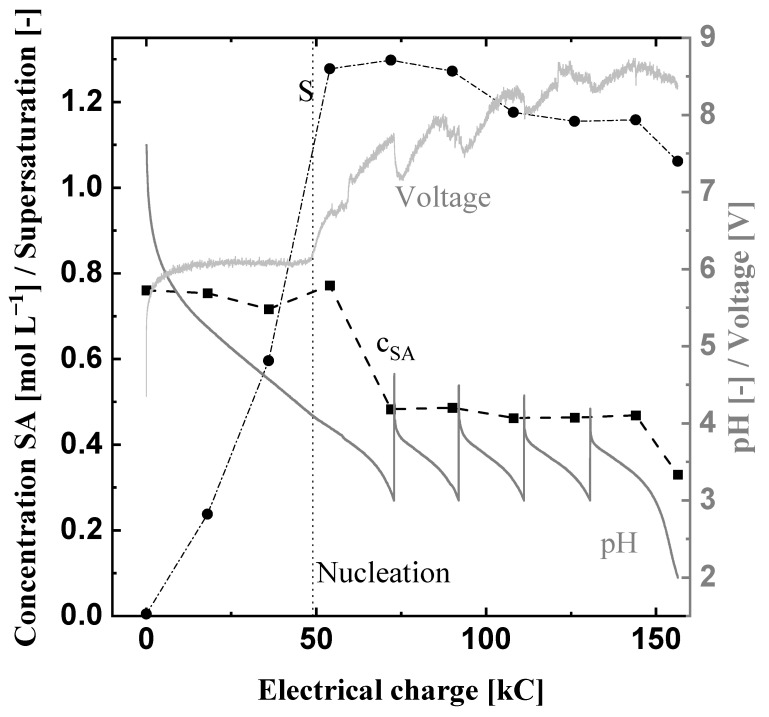
Electrochemical pH-shift crystallization in the prototype at 10 °C, 0.05 A cm^−2^ and a volume flow rate $\dot{V}$ of 0.52 L min^−1^ (Exp3). The black squares are the measured succinic acid concentration, the black cycles show the calculated supersaturation ratio S, and the two gray lines display the course of the pH value and voltage of Exp3.

Alternatively, the voltage and energy requirement can be reduced by decreasing the applied current density. Therefore, the applied current density was cut in half for Exp3. Figure 7 shows the measured succinic acid concentration, pH value, voltage, and the calculated supersaturation ratios of Exp3. The nucleation occurs after about 50 kC, comparable to Exp1. However, since the current density is lower, the nucleation took place after about 2.8 h instead of 1.4 h in Exp1. When nucleation occurs, the supersaturation ratio is about 1.2, which is lower than in Exp1. This can be explained by the lower driving force (current density) of Exp3, which generates the supersaturation more slowly and leaves mere time for the reduction of the supersaturation after nucleation. The supersaturation ratio stays between 1.2 to 1.06 while the succinic acid concentration reduces to 0.5 mol L^−1^ and finally reaches a value of 0.33 mol L^−1^. The lower current density of 0.05 A cm^−2^ leads also to lower supersaturation at the end of the experiment underlining that the crystallization has more time to reduce the slower generated supersaturation. The protonation efficiency is comparable to Exp1 and Exp2 (Table 5). The initial voltage of about 6 V, which is more than two volts less compared to the doubled current density of Exp1, rises to a final value of about 8.5 V.

Nevertheless, the necessary energy for the electrochemical pH-shift crystallization was reduced by approx. 15% to 0.32 kWh compared to Exp1. Exp3 produces 0.53 mol succinic acid crystals with a mean size of 645 μm (compare Supplementary Materials). No direct conclusions regarding the predominant crystallization mechanism can be drawn since breakage due to pumping and agglomeration while drying could not be prevented. With the produced succinic acid, the specific energy demand improved to a value of 0.6 kW h mol^−1^. However, this reduction of the specific energy demand came along with an increased production time of more than 8.5 h, leading to a productivity of 0.06 mol L^−1^ h^−1^. Hence, the operating costs for the production of bio-based succinic acid with electrochemical pH-shift crystallization will be a trade-off between the specific energy demand and the productivity.

**Figure 8:**
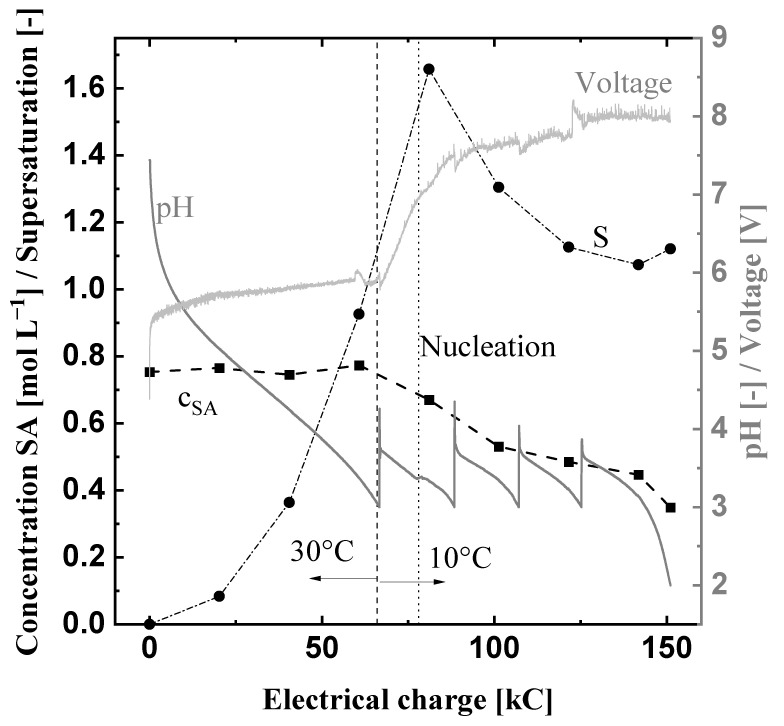
Electrochemical pH-shift crystallization in the prototype at 30 to 10 °C, 0.075 A cm^−2^ and a volume flow rate $\dot{V}$ of 0.52 L min^−1^ (Exp4). The black squares are the measured succinic acid concentration, the black cycles show the calculated supersaturation ratio S and the two gray lines display the course of the pH value and voltage of Exp4.

The aim of Exp4 (Figure 8) is to use the findings of Exp1–3 in order to further reduce the required energy demand within a reasonable production time. Therefore, a current density of 0.075 A cm^−2^ was chosen while the solution was cooled from 30 °C to 10 °C after the first dosage into the anolyte, after approx. 67 kC, which equals about 2.5 h (Figure 8). The succinic acid concentration remains constant until the cooling starts. Thereafter, the concentration decreases to about 0.35 mol L^−1^. The course of the pH value shows a small increase between the first and the second dosage of pH-neutral succinic acid solution, at 76 kC, due to the nucleation. The supersaturation ratio stays below one until the cooling starts. Thereafter, the simultaneous cooling and acidification lead to high supersaturation ratios, which rise fast to a value over 1.6 and are reduced to about 1.1 at the end of the experiment. Exp4 reaches the highest supersaturation ratio, which induces a strong nucleation event. The protonated succinic acid crystallizes and reduces the amount of H^+^ ions in the solution, which leads to a small increase in the pH value. The initial voltage is low at around 5.5 V, as intended. Due to the cooling of the solution and the crystallization, the voltage strongly increases until it reaches values of about 8 V. Nevertheless, the energy demand for Exp4 was successfully reduced to 0.28 kWh. Due to a lower recovery of solid succinic acid crystals ( 0.45 mol), the specific energy demand is higher than in Exp3 (Table 5); however, the succinic acid was produced in three quarters of the time needed in Exp3, leading to a productivity of 0.08 mol L^−1^ h^−1^. The recovery can be increased further if the final operating point (pH = 2 and T = 10 °C) is held in order to reach equilibrium and decrease the final supersaturation.

Assuming that the recovery can be improved by decreasing the final concentration close to the solubility concentration (Figure 4) and taking the low energy demand from Exp4, a theoretical specific energy demand of 0.56 kW h mol^−1^ can be reached. Compared to the energy demand of 1.312 kW h mol^−1^ calculated by Morales et al. [12] for an electrodialysis process consisting of three electrolysis modules, the energy demand is cut in half. This theoretical energy demand for the electrochemical pH-shift crystallization leads to expected electricity costs of about 34 € kmol^−1^, assuming an industrial electricity price of 0.06 € kW^−1^ h^−1^ [17]. Comparing these operational costs expected for electricity with the material costs for pH-adjustment by H_2_SO_4_ (0.14 € kg^−1^) and NaOH (0.3 € kg^−1^) addition [17], which amounts to 38 € kmol^−1^, the electrochemical pH-shift crystallization of bio-based succinic acid shows promising economical potential. However, the operational costs depend strongly on the electricity price and, for a detailed assessment of the potential, the capital costs of the electrolysis module, electrodes, membranes, etc. must be taken into account.

The performance parameters in Table 5 lead to a range of the productivity of the electrochemical succinic acid crystallization between 0.04 mol L^−1^ h^−1^ and 0.13 mol L^−1^ h^−1^. The productivity for the microbial succinic acid production is reported between 6.27 × 10^−3^ mol L^−1^ h^−1^ and 0.09 mol L^−1^ h^−1^ [26]. Since the productivity of the electrochemical crystallization is higher than the biotechnological production rate of succinic acid, small volume electrolysis modules could be used for in situ separation of succinic acid larger fermentation processes [10,11,19]. By adjusting the volume flows or dosages of the fermentation broth in order to control the pH inside the electrolysis module at a value of approx. three, succinic acid crystallization can be operated continuously. Furthermore, the productivity of the electrochemical crystallization can be adjusted flexibly, by controlling the current density, to match the productivity of the fermentation process.

**Figure 9:**
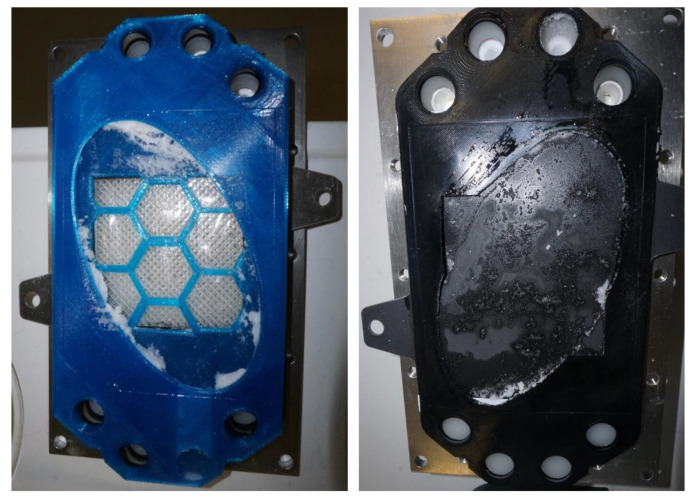
On the left, the prototype of the anode chamber and the membranes are shown after experimentation; on the right, the anode can be seen. No scaling on either the membrane or the anode was observed.

The displayed experiments show that the designed prototype of the anode chamber is capable of producing succinic acid within the electrolysis module for periods of more than 8 h without blocking or scaling on the anode or membrane (Figure 9) at high current densities and high solid loadings (>5 wt%). In Figure 9 the prototype of the anode, the membrane, and the anode after experimentation can be seen. After the suspension is pumped out of the anode chamber, the electrolysis cell is opened. Residual succinic acid crystals are stuck to the walls close to the inlet and outlet of the anode chamber. Similar to the anode, where the remaining crystals are stuck close to the inlet on the bottom but no crystals are stuck to the anode surface. These remaining crystals can easily be removed. However, further optimization of the prototype as well as the entire electrolysis cell can most likely lead to even lower cell voltages and render the electrochemical pH-shift crystallization even more competitive.

## 4. Conclusions

The presented results demonstrate the potential of electrochemical pH-shift crystallization for succinic acid. The prototype of the anode chamber enables the production of crystalline succinic acid within the 100 cm${}^{2}$ electrolysis module of Electrocell (Electro MP Cell^®^). Furthermore, the developed prototype allows to operate and to evaluate the electrochemical crystallization of succinic acid at industry-relevant operation conditions, which are high current densities, the resulting voltage, and high crystal content in the solution. Despite the high current densities and high crystal content in the solution, no blocking of the prototype nor scaling on the anode or membrane was observed.

Furthermore, it was shown that higher temperatures are beneficial in order to reduce energy demand. However, low temperatures and pH values are required at the end of the crystallization process in order to maximize the production of succinic acid crystals. Hence, a cooling profile during the pH-shift crystallization was established. Additionally, the final operating conditions should be held to increase the solid yield since the crystallization kinetics are slower than the supersaturation generation at high current densities. The evaluation of the electrochemical pH-shift crystallization demonstrated that the electron efficiency is high for all investigated temperatures and current densities. Furthermore, it was shown that minimizing operating costs for the electrochemical pH-shift crystallization will be a trade-off between the specific electric energy demand and the productivity.

By adding pH-neutral succinic acid solution, representing a fermentation broth, the supersaturation of the pH-shift crystallization is controlled to values below 1.6. In future works, the dosing strategy together with the cooling profile can be adjusted and refined in order to perform a continuous electrochemical pH-shift crystallization and continuous succinic acid separation.

## Figures and Tables

**Table 1:** Chemicals used for the solubility measurements and the crystallization experiments.

Chemicals	Purity	CAS-Number	Supplier
Succinic acid	≥99%	110-15-6	Carl Roth
Disodium succinate	≥96%	150-90-3	Merck KGaA
Distilled Water	≤0.7 μS cm^−1^	7732-18-5	Intern *
Disodium sulfate	≥99%	7757-82-6	VWR chemicals
Sodium hydroxide	≥99%	1310-73-2	VWR chemicals

* Distillation MonoDest3000 by Lenz Glas Instrumente, Germany.

**Table 2:** Composition and quantity of anolyte and catholyte at the beginning of the crystallization experiments.

Solution	Succinic Aicd	Disodium Succinate	Disodium Sulfate	Volume
[−]	[mol L^−1^]	[mol L^−1^]	[mol L^−1^]	[L]
Anolyte	0	0.78	0.5	0.5
Catholyte	0.42	0	0.5	1

**Table 3:** Operating conditions of the electrochemical pH-shift crystallization experiments.

Name	Current Density	Temperature	$\dot{V}$
[−]	[A cm^−2^]	[°C]	[L min^−1^]
Exp1	0.1	10	0.52
Exp2	0.1	30	0.52
Exp3	0.05	10	0.52
Exp4	0.075	30–10	0.52

**Table 4:** Parameters of the solubility curve for succinic acid in aqueous Na_2_SO_4_ solution fitted to Equation (12).

Temperature	$A_{\mathrm{SOL}}$	$B_{\mathrm{SOL}}$	$C_{\mathrm{SOL}}$	$R^{2}$
[°C]	[mol L^−1^]	[−]	[mol L^−1^]	[−]
10	$3.28\times 10^{-4}$	1.73	0.3	0.89
30	$7.84\times 10^{-5}$	2.3	0.65	0.99

**Table 5:** Comparison of key performance parameters of the showcased experiments.

Name	Produced SA	Final Supersat.	Efficiency	Sp. Energy Demand	Duration	Productivity
[−]	[mol]	[−]	[%]	[kW h mol^−1^]	[h]	[mol L^−1^ h^−1^]
Exp1	0.55	1.19	98	0.69	4.3	0.13
Exp2	0.15	1.04	99	1.89	4.2	0.04
Exp3	0.53	1.06	97	0.6	8.6	0.06
Exp4	0.45	1.12	100	0.62	5.6	0.08

## Supplementary Materials

The following supporting information can be downloaded at: https://www.mdpi.com/article/10.3390/ma15238412/s1, Figure S1: Crystal size distribution of the four experiments; Figure S2: Images of the recovered succinic acid crystals from Exp4 taken with a Table Top TM 3030Plus electron microscope by Hitachi. Figure S3: Raman spectrum of the electrochemically produced succinic acid and bought succinic acid with a purity of 99 %; Table S1: Comparison of the produced SA and its particle size.
