# Supplementary material for: Evaluation of a Prototype for Electrochemical pH-Shift Crystallization of Succinic Acid
Source: Materials (Basel). 2022 Nov 25;15(23):8412. doi: 10.3390/ma15238412 (PMC9738731; doi:10.3390/ma15238412)
Supplement: Supplementary file 1 [file materials-15-08412-s001.zip › materials-1973770-supplementary.pdf]

materials

Supplementary Materials

Figure S1 displays the crystal size distributions of Exp1–Exp4. All distributions show **a** maximum between and between 250 µm and 350 µm. However, these distributions do not lead to a direct conclusion regarding the crystallization process because breakage during the pumping and agglomeration during drying could not be avoided. The produced crystals of Exp4 are exemplary and are highlighted in Figure S2. Furthermore, the Raman spectrum is shown for the produced succinic acid and compared to the spectrum of bought succinic acid with a purity of 99% Figure S12. No additional peaks are visible for the electrochemically produced succinic acid which proves that succinic acid was produced。

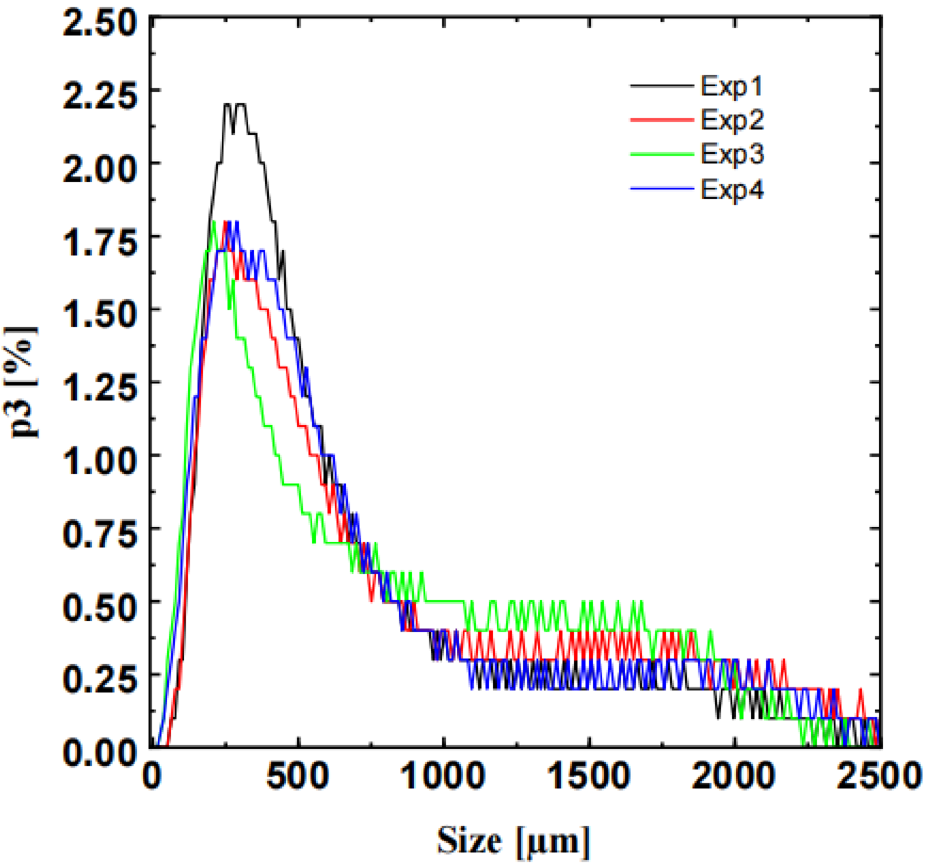

Figure S1. Crystal size distribution of the four experiments.

Table S1. Comparison of particle size.

Name<br>[−]	Produced SA<br>[mol]	X_{50}<br>[µm]
Exp1	0.55	478
Exp2	0.15	595
Exp3	0.53	645
Exp4	0.45	533

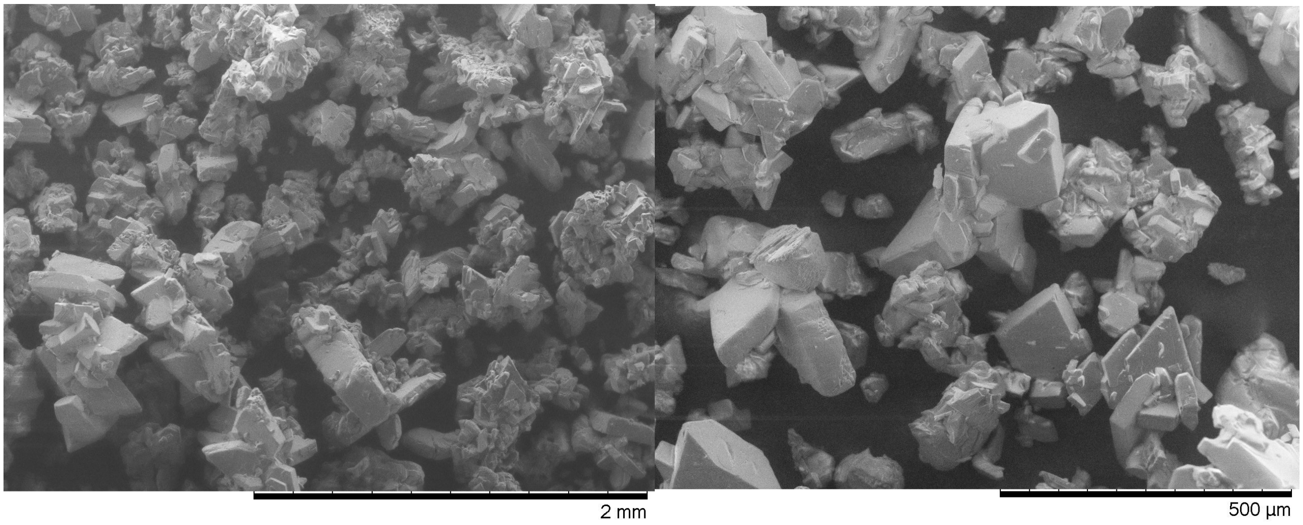

Figure S2. Images of the recovered succinic acid crystals from Exp4 taken with a Table Top TM 3030Plus electron microscope by Hitachi.

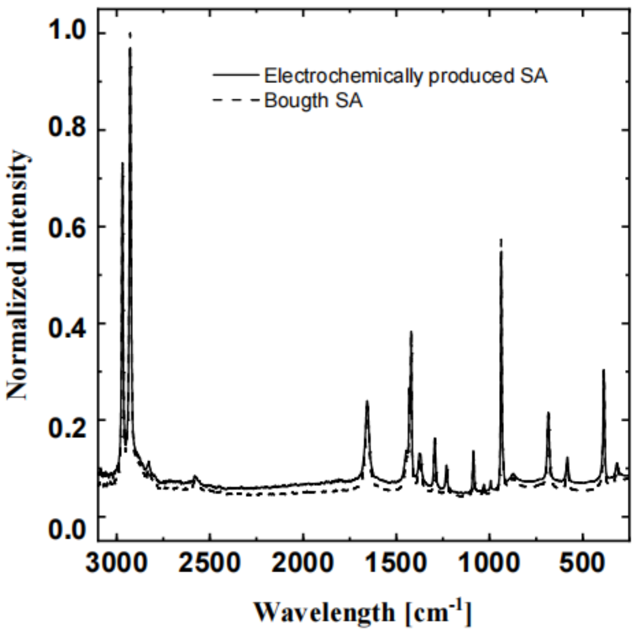

Figure S3. Raman spectrum of the electrochemically produced succinic acid and bought Scheme 99%.
